# Body image concerns in long-term head and neck cancer survivors: prevalence and role of clinical factors and patient-reported late effects

**DOI:** 10.1007/s11764-022-01311-y

**Published:** 2022-12-13

**Authors:** Anna Ivanova, Rubén Rodríguez-Cano, Ingela Lundin Kvalem, Diana Harcourt, Cecilie E. Kiserud, Cecilie D. Amdal

**Affiliations:** 1 Department of Psychology, University of Oslo, Blindern, PB 1094, 0317 Oslo, Norway; 2 PROMENTA Research Center, Department of Psychology, University of Oslo, Oslo, Norway; 3 Centre for Appearance Research, University of the West of England, Bristol, UK; 4 Department of Oncology, Oslo University Hospital, Oslo, Norway; 5 Research support services, Oslo University Hospital, Oslo, Norway

**Keywords:** Body image, Head and neck cancer, Late effects, Quality of life

## Abstract

**Purpose:**

Head and neck cancer (HNC) patients are at risk of long-term body image distress (BID). We aimed to investigate the severity of BID in long-term HNC survivors and to explore the associations between sociodemographic and clinical factors, patient-reported late effects, and cancer-related body image (BI) concerns.

**Methods:**

This cross-sectional study included quality of life and BI assessment in an 8-year (SD = 1.58) follow-up after treatment among 258 HNC survivors. Multinomial logistic regression analysis was used to investigate the relationship between three groups of BI concerns (no concerns, mild to moderate concerns, and BID) and patient-reported late effects. Sociodemographic and clinical variables were included in the model as covariates.

**Results:**

A total of 51.2% of participants had mild to moderate BI concerns, and 9.5% reported BID. Compared to those with no BI concerns, participants with BID were more likely to live without a partner, to have had radiotherapy and surgery, and to report worse emotional functioning and higher oral and throat pain. Compared to participants with no BI concerns, those with mild to moderate concerns reported higher oral and throat pain and speech problems.

**Conclusions:**

Some level of cancer-related BI concerns persisted in the majority of HNC survivors many years after treatment, while a small proportion of survivors experienced BID. BI concerns were associated with treatment modality and patients’ daily functioning and symptoms.

**Implications for Cancer Survivors:**

Insight into factors associated with BI problems may help to identify survivors at risk and may facilitate closer follow-up of survivors in need.

## Introduction

Head and neck cancer (HNC) patients are at risk of body image distress (BID) due to significant changes in physical appearance and body functioning [1–6]. Surgical treatment in the head and neck area can lead to disfigurement and scarring, while radiotherapy often causes fibrosis and swelling and can result in a number of functional changes that persist over time [3]. Pain, fatigue, and oral late effects such as xerostomia, dental problems, trismus, dysphagia, and mucositis are among the symptoms that have been shown to affect patients’ body image [3] and their overall quality of life [7–10]. Loss of functionality may affect how individuals perceive their bodies, leading to upward comparisons with able-bodied individuals and the former self, as well as feelings of self-consciousness and shame, which can further exacerbate BID [11]. Depressive symptoms and psychological distress following diagnosis and treatment for HNC have also been associated with negative body image [12, 13].

Cancer-related body image (BI) concerns appear to rise temporarily following HNC diagnosis and treatment [2] and tend to gradually decline [14, 15]; however, some patients may experience persistent BID [6, 16]. Melissant et al. [6] reported a 17–20% BID prevalence in patients with a short to median time since treatment of 3.3–3.5 years. Little is, however, known about the prevalence of and psychosocial and clinical factors associated with BID in HNC survivors with longer periods of time after treatment (e.g., more than 5 years). Assessing persistent BID, as well as central predicting factors, in HNC survivors would help to inform patients and help clinicians address and prevent possible long-term negative psychosocial consequences.

Using a sample of 258 patients with an average follow-up of 8 years after treatment, we aimed (1) to investigate the severity of BI concerns in long-term HNC survivors and (2) to explore the associations between sociodemographic factors, clinical factors (i.e., cancer subsite, cancer stage, and type of treatment), patient-reported late effects (emotional functioning, physical functioning, fatigue, and oral symptoms), and persisting cancer-related BI concerns.

## Methods

### Study design and participants

The current study is a cross-sectional secondary analysis from the “H&N Cancer (HNC); Survivorship and Late Effects” study (ClinicalTrials.gov Identifier: NCT04758026), conducted at Oslo University Hospital (OUH), Norway, from October 2018 to October 2020. In total, 522 survivors diagnosed with HNC and treated at OUH were invited to participate by mail, and 280 consented to participate in the study. Inclusion criteria were histologically or cytologically verified invasive carcinoma of the head and neck region diagnosed between 2006 and 2012, ability to understand and respond to the questionnaires, and ability to attend the clinical examination. Exclusion criteria were unwillingness to answer the questionnaires, ongoing treatment for second primary cancer, or relapse at the time of the survey. Eligible survivors completed a set of validated questionnaires on paper at home before attending a 1-day in-person visit at OUH. Out of 280 participants, 265 completed the hospital visit and questionnaires. Seven participants were subsequently excluded from this data analysis: two participants had not received radiotherapy treatment, three received the diagnosis and treatment before the age of 18, and two received recent treatment after cancer recurrence. The time from treatment to survey was more than 5 years for all participants.

### Measures

#### Body image concerns

The outcome measure, BI concerns, was assessed with the *Body Image Scale* (BIS) (*α* = 0.92) [17]. The BIS is a 10-item self-report scale developed to measure body image changes in cancer patients. The BIS has shown sound validity and reliability [17] and has been used in research on HNC patients [5, 14–16, 18–20]. Response categories ranged from “not at all” (0) to “very much” (3). The total score ranges from 0 to 30, where a higher score represents more BI concerns and ultimately BID with a cutoff of BIS ≥ 10 [14, 21, 22]. Based on the clinical relevance, the total score was divided into three groups for further analysis: “No BI concerns” [BIS = 0], “Mild to moderate BI concerns” (0 < BIS < 10), and “BID” (BIS ≥ 10) [21, 23].

#### Patient-reported late effects

Patient-reported late effects included self-reported levels of functioning and symptoms. Functional characteristics were assessed with the *European Organization for Research and Treatment of Cancer core quality of life questionnaire*, EORTC QLQ-C30 version 3 [24], and symptoms were assessed using the HNC-specific module, EORTC QLQ-H&N35 [25]. The current study included subscales on physical functioning (5 items) and emotional functioning (4 items) from the EORTC QLQ-C30, as well as oral symptoms subscales from the EORTC QLQ-H&N35 (i.e., subscales on pain (4 items), swallowing (4 items), speech problems (3 items), teeth (1 item), mouth opening (1 item), dry mouth (1 item), and sticky saliva (1 item)). Response categories ranged from “not at all” (1) to “very much” (4). All responses were converted to 0–100 subscale scores, where a higher score represents a higher degree of function or a higher degree of problems. Based on recommendations in the literature [26] for the EORTC QLQ-C30 and QLQ-H&N35, a difference of ≥ 10 is regarded as a clinically significant difference between groups.

Fatigue was measured using the *Fatigue Questionnaire* (FQ) [27]. The FQ contains 11 items concerning physical and mental fatigue during the last month. Response options ranged from “better than usual” (0) to “much worse than usual” (3). The responses were combined into a total fatigue score with a maximum score of 33 [27].

#### Sociodemographic and clinical variables

Sociodemographic and clinical data were collected with a clinical report form during the hospital visit. Data on medical history were collected from the patients’ medical records. Sociodemographic variables included age, gender, level of education, living with or without partner, and employment status. Employment status was divided into two groups for further analysis: “on disability” and “not on disability”. Clinical variables included tumor location, cancer stage, and cancer treatment. Cancer treatment was divided into two groups for further analysis: the “radiotherapy” group, which included those who received radiotherapy ± chemotherapy, and the “radiotherapy and surgery” group, which included those who received surgery and radiotherapy ± chemotherapy.

### Statistical analysis

Descriptive statistics (proportions for categorical variables and mean [SD] for continuous variables) were calculated. We assessed the differences in sociodemographic factors, clinical factors, and patient-reported late effects (functioning and oral symptoms) by the BI concern groups. For categorical variables, we used chi-square tests, and for continuous variables, we used Kruskal‒Wallis tests. To analyze the predictors of BI concerns, we ran a sequential multinomial logistic regression analysis. The first step included sociodemographic and clinical characteristics. The second step added patient-reported late effects that were statistically significant in the bivariate analyses using forward entry. We ran the analyses with SPSS version 28 and used *p* < 0.05 for the significance level.

## Results

### Sample characteristics

The final sample consisted of 258 participants (66.7% male; *M age* = 64.99, SD = 8.92 years). The sample characteristics are presented in Table 1. The most common tumor locations were the oropharynx (53.1%) and oral cavity (17.4%), and the majority (51.9%) of patients had stage IV cancer. Two-thirds (66.3%) of the sample was treated with both radiotherapy (either chemoradiotherapy or radiotherapy) and surgery, and the rest was treated with radiotherapy (either radiotherapy monotherapy or chemoradiotherapy). The mean time since treatment was 8.26 years, *SD* = 1.58 (range: 6.00–12.00 years). Approximately 50% of the patients had mild to moderate BI concerns, while 9% reported BID (Table 1).

**Table 1:** Characteristics of the sample: sociodemographic, clinical, appearance-related, and functional factors (*N* = 258)

**Sociodemographic characteristics**	***Number***	***Percent***
Gender		
Female	86	33.3
Male	172	66.7
Education		
< 10 years	45	17.4
≥ 10 years	213	82.6
Living situation		
With partner	189	73.3
Without partner	69	26.7
Employment status		
On disability	48	18.6
Employed	97	37.6
Retired	106	41.1
Unemployed	4	1.6
On sick leave	3	1.2
Age (years)		
Mean [SD]		64.99 [8.92]
**Clinical characteristics**		
Tumor location		
Oral cavity	45	17.4
Oropharynx	137	53.1
Larynx	17	6.6
Nasopharynx	7	2.7
Hypopharynx	4	1.6
Unknown primary	12	4.7
Other HNC	36	14
Morphology		
Squamous cell carcinoma	218	84.5
Adenocarcinoma	4	1.6
Mucoepidermoid carcinoma	5	1.9
Adenoid cystic carcinoma	7	2.7
Acinic cell carcinoma	4	1.6
Invasive ductal carcinoma	4	1.6
Undifferentiated carcinoma	8	3.1
Cancer stage (UICC)		
I	37	14.3
II	38	14.7
III	48	18.6
IV	134	51.9
Treatment type		
Radiotherapy monotherapy	21	8.1
Chemoradiotherapy	66	25.6
Surgery + chemoradiotherapy	69	26.7
Surgery + radiotherapy	102	39.5
Body image distress (BID)		
No concerns (BIS = 0)	102	39.5
Mild to moderate (0 < BIS < 10)	132	51.2
BID (BIS ≥ 10)	24	9.3
Years after treatment		
Mean [SD]		8.26 [1.58]
**Patient-reported late effects (scale scores)**	***Mean***	***SD***
Physical functioning (0–100)	82.07	21.54
Emotional functioning (0–100)	82.21	20.92
Pain (0–100)	19.50	20.86
Swallowing difficulties (0–100)	18.15	21.73
Speech problems (0–100)	15.25	19.99
Teeth problems (0–100)	31.78	35.20
Opening mouth (0–100)	26.98	34.46
Dry mouth (0–100)	57.88	34.92
Sticky saliva (0–100)	48.51	35.22
Fatigue (0–33)	14.65	5.73
BIS (0–30)	3.39	4.90

*BID* body image distress, *BIS* Body Image Scale, *HNC* head and neck cancer, *RT* radiotherapy, *UICC* Union for International Cancer Control

### Bivariate analyses

Tables 2 and 3 present the results of the bivariate analyses. From chi-square tests (Table 2), significant differences in BI concerns were found for living with partner (*χ*^2^(2) = 8.62, *p* = 0.013) and being on disability (*χ*^2^(2) = 14.91, *p* < 0.001). Gender and treatment type did not differ significantly between the groups but were included in the multinomial logistic regression since the analysis revealed associations at *p* < 0.10. The Kruskal‒Wallis tests (Table 3) revealed statistically significant differences in all patient-reported late effects across the three BI concerns groups. Pairwise comparisons with adjusted *p* values showed significant differences in all patient-reported late effects between those with no BI concerns and those with BID, as well as between those with no BI concerns and those with mild to moderate concerns. There were no significant differences in physical function, oral and throat pain, swallowing difficulties, or speech problems between the mild to moderate BI concerns and BID groups. Clinically significant differences (difference in scores of > 10 [26]) were found between those with no BI concerns and those with BID for all of the patient-reported late effects, apart from fatigue.

**Table 2:** Group differences in those who reported no BI concerns, mild to moderate BI concerns, and BID using chi-square tests (for categorical variables)

Variable	No BI concerns (*n* = 102)	Mild to moderate BI concerns (*n* = 132)	BID (*n* = 24)	*χ*^2^	*Cramer’s V*
	*n (%)*	*n (%)*	*n (%)*		
Gender				5.47	
Male	75 (73.5)	85 (64.4)	12 (50.0)		
Female	27 (26.5)	47 (35.6)	12 (50.0)		
Education				0.56	
< 10 years	20 (19.6)	21 (15.9)	4 (16.7)		
≥ 10 years	82 (80.4)	111(84.1)	20 (83.3)		
Lives with partner				8.62^**^	.18
Yes	81 (79.4)	96 (72.7)	12 (50.0)		
No	21 (20.6)	36 (27.3)	12 (50.0)		
On disability				14.91^***^	.24
Yes	12 (11.8)	25 (18.9)	11 (45.8)		
No	90 (88.2)	107 (81.1)	13 (54.2)		
Treatment type				5.48	
Radiotherapy	38 (37.3)	46 (34.8)	3 (12.5)		
Radiotherapy and surgery	64 (62.7)	86 (65.2)	21 (87.5)		
Cancer types				7.34	
Oral cavity	14 (13.7)	25 (18.9)	6 (25.0)		
Oropharynx	60 (58.8)	69 (52.3)	8 (33.3)		
Larynx	4 (3.9)	10 (7.6)	3 (12.5)		
Other HNC	24 (23.5)	28 (21.2)	7 (29.2)		
Cancer stages (UICC)				1.25	
I–II	26 (25.5)	41 (31.1)	8 (34.8)		
III–IV	76 (74.5)	91 (68.9)	15 (65.2)		

Effect sizes (Cramer’s *V*) are reported for statistically significant results

*BI* body image, *BID* body image distress, *χ*^2^ chi-square test statistic

^**^p < .01; ***p < .001

**Table 3:** Group differences by body image concerns

Variable	No BI concerns (*n* = 102)	Mild to moderate BI concerns (*n* = 132)	BID (*n* = 24)	*H*	*Significant pairwise differences*	*r*
	*Mean [SD]*	*Mean [SD]*	*Mean [SD]*			
Age	65.06 [8.87]	65.23 [9.04]	63.38 [8.61]	1.03		
Physical function	87.97 [19.20]	80.10 [21.16]	67.78 [24.99]	25.22***	BID-NC*** MC-NC***	.38 .25
Emotional function	89.87 [17.06]	80.24 [20.20]	60.42 [22.56]	42.39***	BID-MC*** BID-NC*** MC-NC***	.31 .54 .27
Oral and throat pain	11.14 [14.17]	23.25 [21.76]	34.37 [25.69]	30.14***	NC-MC*** NC-BID***	.29 .40
Swallowing problems	12.50 [18.00]	20.96 [22.50]	26.74 [26.58]	15.58***	NC-MC** NC-BID**	.22 .27
Speech problems	8.06 [13.73]	18.77 [21.14]	26.39 [25.76]	27.97***	NC-MC*** NC-BID***	.29 .37
Teeth problems	21.78 [29.98]	36.87 [36.67]	45.83 [37.83]	14.12***	NC-MC*** NC-BID***	.26 .26
Opening mouth	18.81 [29.22]	30.30 [36.01]	43.06 [38.67]	11.45***	NC-MC* NC-BID***	.20 .27
Dry mouth	48.37 [34.67]	62.88 [33.37]	70.83 [35.86]	13.85***	NC-MC*** NC-BID**	.26 .26
Sticky saliva	38.56 [34.71]	53.69 [34.24]	62.50 [33.06]	15.16***	NC-MC*** NC-BID***	.27 .27
Fatigue	12.94 [4.70]	14.97 [5.60]	20.21 [6.75]	30.76***	NC-MC** NC-BID*** MC-BID**	.22 .47 .27

*BI* body image, *BID* body image distress, *MC* mild to moderate concerns, *NC* no concerns, *SD* standard deviation, *H* Kruskal‒Wallis test statistic, *r* effect sizes for pairwise comparisons for significant results

^*^p < .05; **p < .01; ***p < .001

### Predictors of body image concerns

Multinomial logistic regression analyses are displayed in Table 4. The first-step model showed good fit (*χ*^2^(418) = 396.14, *p* = 0.772 *R*^2^_Nalgerkerke_ = 0.16). Compared to patients without BI concerns, participants with BID were more likely to live without a partner, be on disability, and have received radiotherapy and surgery treatment. Compared to those in the mild to moderate concerns group, patients with BID were more likely to live without a partner, be on disability, and have received both radiotherapy and surgery. There were no significant differences when mild to moderate BI concerns were compared with no BI concerns.

**Table 4:** Multinomial logistic regression predicting body image concerns group membership

Variable	No BI concerns^a^ vs. mild to moderate		No BI concerns^a^ vs. BID		Mild to moderate^a^ vs. BID	
	*OR*	*95% CI for OR*	*OR*	*95% CI for OR*	*OR*	*95% CI for OR*
Model 1 (*R*^*2*^ = .16)^b^						
Age	1.01	[0.98; 1.05]	1.02	[0.96; 1.09]	0.99	[0.93; 1.05]
Gender: Female [0] vs. male [1]	0.70	[0.39; 1.26]	0.45	[0.16; 1.26]	0.65	[0.25; 1.71]
Education: < 10 years [0] vs. ≥ 10 years [1]	1.51	[0.73; 3.13]	1.45	[0.37; 5.72]	0.96	[0.25; 3.62]
Lives with partner: No [0] vs. Yes [1]	0.67	[0.36; 1.26]	0.18^**^	[0.06;0.52]	0.27^*^	[0.10; 0.73]
On disability: No [0] vs. Yes [1]	1.84	[0.83; 4.08]	6.77^**^	[2.10; 21.79]	3.68^*^	[1.28; 10.55]
Cancer type						
Oral cavity [0] vs. oropharynx [1]	0.63	[0.26; 1.55]	0.29	[0.06; 1.33]	0.46	[0.11; 1.92]
Oral cavity [0] vs. larynx [1]	1.26	[0.30; 5.26]	1.75	[0.18; 16.71]	1.38	[0.18; 10.69]
Oral cavity [0] vs. other [1]	0.60	[0.24; 1.48]	0.56	[0.13; 2.34]	0.93	[0.24; 3.56]
Cancer stage: I-II [0] vs. III-IV [1]	0.99	[0.49; 2.01]	1.65	[0.49; 5.56]	1.66	[0.53; 5.25]
Treatment: RT [0] vs. RT + S [1]	1.10	[0.61; 1.99]	5.21^*^	[1.19; 22.88]	4.75^*^	[1.12; 20.13]
Model 2 (*R*^*2*^ = .34)^b^						
Emotional function	0.98	[0.97; 1.00]	0.95^***^	[0.92, 0.97]	0.96^**^	[0.93, 0.99]
Oral and throat pain	1.02^*^	[1.005; 1.04]	1.05^**^	[1.02; 1.08]	1.02	[1.00; 1.05]
Speech problems	1.02^*^	[1.003; 1.05]	1.00	[0.97; 1.04]	0.98	[0.95; 1.01]

*BID* body image distress, *RT* radiotherapy, *RT* + *S* radiotherapy and surgery

^*^p < .05; **p < .01; ***p < .001

^a^Reference category

^b^Pseudo R-Square (Nagelkerke)

The second step included patient-reported late effects. The model presented a good fit and doubled the explained variance in BI concerns (*χ*^2^(480) = 386,27, *p* = 0.999, *R*^2^_Nalgerkerke_ = 0.34). Relative to participants with no BI concerns, those with mild to moderate BI concerns reported higher oral and throat pain and speech problems. Compared to participants with no BI concerns, those with BID were more likely to have worse emotional functioning and higher oral and throat pain. Finally, compared to patients with mild to moderate BI concerns, participants with BID had worse emotional function. Being on disability was no longer a significant predictor of BI concerns when patient-reported late effects were added to the model.

## Discussion

To our knowledge, this is the first study to investigate prevalence rates and potential demographic, clinical, and functional predictors of BI concerns in long-term HNC survivors. Specifically, we examined associations between BI concerns (no concerns, mild to moderate concerns, and BID) and common late effects of HNC and its treatment, as well as clinical factors. In multivariable analysis, patients with BID (compared to no cancer-related BI concerns) were more likely to live without a partner, be on disability, have received a combination of radiotherapy and surgery, have worse emotional functioning, and have higher oral and throat pain. Those with mild to moderate BI concerns, compared to survivors without BI concerns, reported more oral and throat pain and more speech problems.

The prevalence of BID in our study was lower than the rates identified in the literature review by Rhoten et al. [3] (25–77%). This may be due to differences in body image instruments used in the reviewed studies, as well as differences in the length of follow-up, which in the reviewed studies ranged between immediately after treatment to more than a year. Our scores are also lower than those in other studies that have used BIS [6, 14–16, 19, 28]. Graboyes et al. [14] reported BID rates of 11% preoperatively and 25% and 27% at 1 and 3 months posttreatment, respectively, while Melissant et al. [6] found a 13% prevalence rate of BID when using the BIS ≥ 10 cutoff in their sample. Other studies reported a mean BIS score of 4.50–4.93 [16, 19] and a median of 4–6 [15, 19], which are higher than the scores in our sample. This was expected, as BI concerns have been shown to gradually decrease posttreatment, since patients adapt to changes in their appearance and functioning with time [15]. Our findings, however, indicated that some level of cancer-related BI concerns persisted in the majority of survivors many years after treatment, while a small proportion of survivors continue to experience serious cancer-related BI concerns that can be classified as BID.

Our findings showed that patients with BID, compared to those with no BI concerns, were more likely to have undergone both radiotherapy and surgical treatment. There were no associations between mild to moderate BI concerns and treatment modality. Surgical treatment in such highly visible areas of the body as the face and neck leads to noticeable changes in appearance, which in turn can negatively affect body image satisfaction. Hung et al. [20] identified radical surgery to the face and neck areas as the strongest predictor of worse body image outcomes in head and neck cancer patients in their study, with the worst outcomes experienced by patients who had the most extensive surgeries. Previous research also indicated that patients treated with both surgery and radiotherapy experienced higher levels of disfigurement [28]. Since patients with higher disfigurement are more likely to have serious BI concerns, this may explain why the association was significant for the BID group.

Future research should also consider the role of potential moderating factors, e.g., investment in appearance, dispositional outlook, and self-esteem [29]. These may be useful when designing pre- and posttreatment interventions.

Our findings were consistent with prior studies that have found that body image in HNC patients can be affected by experiences and perceptions related to body functioning [5]. All patient-reported late effects that were included in bivariate analyses showed significant associations with BI concerns, with pairwise comparisons revealing both clinically and statistically significant differences for all patient-reported late effects between those who had no BI concerns and BID. Adding late effects variables to the multinomial regression model doubled the explained variance in BI concerns.

The significant association between BID and emotional functioning in multivariable analysis in our study is in line with previous research that revealed a positive relationship between depression and BID [12, 13]. However, more research is needed to explore the directionality of this relationship, since patients with BID report high levels of preoccupation with body image changes, as well as disruptions to their behavioral and emotional functioning [2].

Interestingly, our results indicated that oral and throat pain was a significant predictor of both mild to moderate BI concerns and BID (relative to no BI concerns). To our knowledge, our study is the first to report this association. Similarly, pain was positively related to body image dissatisfaction among patients with arthritis [30] and with lymphedema after breast cancer treatment [31]. Sündermann and colleagues [32] suggested that chronic pain may affect body image due to negative perceptions and appraisal of one’s body and its functionality, which creates a negative emotional response. HNC patients report experiencing the highest prevalence of pain compared to other cancer types [33], which may become a persistent reminder of their bodily changes.

We were surprised to find that speech problems were a significant predictor of mild to moderate BI concerns compared to no BI concerns but not of BID in multivariable analysis. Fingeret et al. [5] found that those with speech and eating difficulties exhibited the highest level of body image dissatisfaction compared to those without concerns. Their findings also revealed that those with speech and eating difficulties scored lower on a number of quality of life outcomes, such as physical, emotional, social, and functional well-being [5]. Since patients with BID in our sample generally reported worse functioning and higher symptomatology, it is possible that speech problems in relation to body image were less relevant for them than for patients with mild to moderate BI concerns.

Our results indicated that gender was not a significant predictor of BID. This was unexpected since research on body image and visible difference generally shows that women tend to be consistently more dissatisfied with their body and appearance than men, regardless of age [34]. More men than women in our sample had stage III and IV cancer diagnoses, which could have affected the extent of treatment and in turn led to more changes in functionality and body image. One of the implications of these findings is that male patients, as opposed to the common belief, may be at a similar risk of body image problems posttreatment, as are female patients [16]. This should be considered when offering BID interventions to HNC patients.

Living without a partner was associated with BID compared to no BI concerns. Perhaps living with a partner has a protective role against BID due to social support. Social support is an important coping factor in appearance change adjustment [4].

Being on disability was significantly associated with BI concerns in bivariate analysis but not in the multivariable analysis. While bivariate findings are not the same as multivariable findings, this is worth mentioning, since disability has been associated with more severe BID in previous research [35]. HNC survivors on disability may experience more comorbidities, as well as a higher symptom burden, which in turn may influence their body image. It is, however, possible that body image may also affect people’s return to work [3]. Individuals with high BI concerns may not only be distressed about the loss of their previous appearance and functioning but also feel stigmatized and misjudged and therefore have difficulties returning to work due to fear of negative evaluations [36].

### Practical implications

The findings of the present study contribute to knowledge about long-term BID in HNC survivors, which helps improve the understanding of patients’ needs and can facilitate changes in rehabilitation programs. Currently, there are no effective evidence-based interventions that specifically address BID in HNC patients [1]. More knowledge about long-term BID may increase acceptance and coping among HNC survivors who experience BI problems, as well as make health care professionals more aware of the problem and thereby improve care. Furthermore, insight into factors associated with body image problems will help to identify survivors at risk and may facilitate closer follow-up of survivors in need. Studies are needed to explore interventions that can decrease BID in survivors with this problem.

### Strengths and limitations

The main strengths of this study include the unique focus on HNC survivors on average 8 years after treatment, the large clinical sample size, and the use of validated measures that allow comparison to other studies in the field. The study also has some limitations. It relies on self-report measures, which can be affected by respondent characteristics. The cross-sectional design limits our understanding of the directionality of the relationships between patient-reported late effects and body image concerns. Some researchers have also argued that BIS may not be the ideal measure to assess body image in HNC survivors, since it was designed for patients with breast cancer [23, 35]. However, since the scale has been used successfully with various cancer groups over the years, it is reasonable to suggest that it does identify relevant issues for a cancer population overall. At the same time, since BI concerns have not been previously studied in long-term HNC survivors, it is unclear whether BIS was able to capture their experiences in full.

Future studies may consider the use of newly developed and validated scales designed specifically for HNC patients, such as MBIS-HNC [37] or IMAGE-HN [35]. Longitudinal designs with longer-term follow-up would allow us to understand the changes in BID over time, as well as the long-term consequences of disease and its treatment for patients’ body image and overall quality of life.

## Data Availability

The datasets generated and/or analyzed during the current study are available from the corresponding author on reasonable request.
